# Clinical and electrocardiographic outcomes evaluated by telemedicine of outpatients with clinical suspicion of COVID-19 treated with chloroquine compounds in Brazil^†^

**DOI:** 10.3389/fcvm.2023.1028398

**Published:** 2023-02-15

**Authors:** Bruno R. Nascimento, Gabriela M. M. Paixão, Luìs Antônio B. Tonaco, Ana Carolina D. Alves, David C. Peixoto, Leonardo B. Ribeiro, Mayara S. Mendes, Paulo R. Gomes, Magda C. Pires, Antonio Luiz P. Ribeiro

**Affiliations:** ^1^Serviço de Cardiologia e Cirurgia Cardiovascular e Centro de Telessaúde do Hospital das Clínicas da UFMG, Belo Horizonte, MG, Brazil; ^2^Departamento de Clínica Médica, Faculdade de Medicina da Universidade Federal de Minas Gerais, Belo Horizonte, MG, Brazil; ^3^Curso de Medicina, Faculdade de Saúde e Ecologia Humana (FASEH), Belo Horizonte, MG, Brazil; ^4^Departamento de Estatística, Instituto de Ciências Exatas (ICEX) da Universidade Federal de Minas Gerais, Belo Horizonte, MG, Brazil

**Keywords:** COVID-19, chloroquine, treatment, outcomes, prognosis, electrocardiogram, telemedicine

## Abstract

**Aims:**

To evaluate clinical and electrocardiographic outcomes of patients with COVID-19, comparing those using chloroquine compounds (chloroquine) to individuals without specific treatment.

**Methods:**

Outpatients with suspected COVID-19 in Brazil who had at least one tele-electrocardiography (ECG) recorded in a telehealth system were enrolled in two arms (Group 1: chloroquine and Group 2: without specific treatment) and one registry (Group 3: other treatments). Outcomes were assessed through follow-up calls (phone contact, days 3 and 14) and linkage to national mortality and hospitalization databases. The primary outcome was composed of: hospitalization, intensive care admission, mechanical ventilation, and all-cause death, and the ECG outcome was the occurrence of major abnormalities by the Minnesota code. Significant variables in univariable logistic regression were included in 4 models: 1-unadjusted; 2-adjusted for age and sex; 3-model 2 + cardiovascular risk factors and 4-model 3 + COVID-19 symptoms.

**Results:**

In 303 days, 712 (10.2%) patients were allocated in group 1, 3,623 (52.1%) in group 2 and 2,622 (37.7%) in group 3; 1,969 had successful phone follow-up (G1: 260, G2: 871, and G3: 838). A late follow-up ECG was obtained for 917 (27.2%) patients [group 1: 81 (11.4%), group 2: 512 (14.1%), group 3: 334 (12.7%)]. In adjusted models, chloroquine was independently associated with greater chance of the composite clinical outcome: phone contact (model 4): OR = 3.24 (95% CI 2.31–4.54), *p* < 0.001. Chloroquine was also independently associated with higher mortality, assessed by phone + administrative data (model 3): OR = 1.67 (95% CI 1.20–2.28). However, chloroquine did not associate with the occurrence of major ECG abnormalities [model 3; OR = 0.80 (95% CI 0.63–1.02, *p* = 0.07)]. Abstracts with partial results of this work was accepted in the American Heart Association Scientific Sessions, November 2022, in Chicago, IL, USA.

**Conclusion:**

Chloroquine was associated with a higher risk of poor outcomes in patients suspected to have COVID-19 when compared to those who received standard care. Follow-up ECGs were obtained in only 13.2% of patients and did not show any significant differences in major abnormalities amongst the three groups. In the absence of early ECG changes, other side effects, late arrhythmias or deferral of care may be hypothesized to explain the worse outcomes.

## Introduction

The pandemic of the new coronavirus disease (COVID-19) caused by the SARS-CoV-2 was decreed by the World Health Organization (WHO) on 11 March 2020. Worldwide, as of 23rd December 2022, over 660 million cases and 6.7 million deaths have already been recorded (1). Brazil has been severely hit by the pandemic, ranking fifth in the absolute number of reported cases (over 36.1 million) and second in the number of deaths (over 692 thousand) (1). The Brazilian cumulative incidence rate is now approximately 16,750 cases per 100,000 inhabitants, with an accumulated mortality rate of around 322 deaths per 100,000 inhabitants (2). COVID-19 proved to be a challenging health condition, with high transmissibility, potential systemic involvement, and without well-established treatments. It led to the collapse of several health systems, noticeably in areas with limited health structure in the country (3).

The clinical presentation of COVID-19 is mainly respiratory, with mild involvement in approximately 80% of cases. The new coronavirus also affects the cardiovascular system, especially in severe cases and in patients with established cardiovascular disease. Acute myocardial injury, the most frequent cardiac abnormality (8–20% of patients), is associated with a worse prognosis (4–6). Furthermore, there is an association of COVID-19 with acute coronary syndromes, venous thromboembolism, heart failure—as a consequence of a deterioration of underlying heart disease or induced by viral myocarditis—and a myriad of rhythm disturbances (7). Ventricular tachycardia, a marker of disease severity, is associated with increased serum troponin levels (8) and QT prolongation, observed in approximately 13% of infected patients (8, 9).

Given the absence of well-established pharmacological treatments and the limited knowledge about the natural history of the disease and predictors of worse outcomes—especially in high-risk groups—there were several recommendations for the use of off-label drugs, based on limited scientific evidence, noticeably *in vitro* and observational studies (10, 11). This occurred especially—but not exclusively—in the beginning of the pandemic. Chloroquine compounds (hydroxychloroquine/chloroquine) (namely chloroquine)—associated or not with azithromycin—and ivermectin were among these drug schemes. However, larger-scale observational studies and, more recently, Brazilian randomized trials (12, 13) failed to demonstrate any benefit. Although relatively well tolerated, chloroquine compounds can induce cardiovascular side effects, such as QT interval prolongation and potentially fatal arrhythmias (14, 15), which may further increase the patients’ cardiovascular risk. Epidemiological studies assessing cardiac effects of chloroquine for the treatment of COVID-19, with real-life data and broad inclusion criteria, are still limited. We aimed to evaluate the clinical and electrocardiographic outcomes of outpatients with suspected COVID-19, comparing those using chloroquine with individuals without specific treatment and a parallel registry of individuals using other drug classes.

## Materials and methods

The procedures and methods of this study will be made available for replication upon reasonable request directed to the corresponding author. The Institutional Review Board of Universidade Federal de Minas Gerais approved the study under CAAE number 37228120.9.0000.5149.

This is a comparative observational study with prospective data collection. The sample consisted of two arms and one parallel registry, and clinical and electrocardiographic outcomes were assessed remotely. The project was funded by the Brazilian Ministry of Health and conducted by the Telehealth Center of Hospital das Clínicas, Universidade Federal de Minas Gerais (Belo Horizonte, MG, Brazil). Remote data collection occurred in health units connected to the Teleassistance Network of Minas Gerais (Rede de Teleassistência de Minas Gerais–RTMG), and the tele-electrocardiography (ECG) system for COVID-19, in all Brazilian regions.

During the COVID-19 pandemic, RTMG adapted its mobile ECG application to provide clinical decision support for COVID-19 cases in health units, especially in primary care, with demographic and clinical data collection, and ECGs for remote interpretation. It was recommended by health authorities that an ECG be obtained before and following the initiation of drugs for COVID-19. ECGs were captured by commercial equipment linked to specific proprietary software, which allows for getting the ECG signal and clinical data, and transmitted by internet to a central server at the Telehealth Center. The requesting healthcare provider collected baseline history, demographic and clinical data. ECGs were centrally analyzed by a team of experienced cardiologists, utilizing specific semi-automated software with measurement and magnification tools, with visual inspection and subsequent classification by the Minnesota code. Minnesota is the most widely used ECG classification system in the world, developed in the 1950s by Dr. Henry Blackburn, which utilizes a defined set of measurement rules to assign specific numerical codes according to the severity of findings (16, 17). In the presence of a discrepancy between automated reports and the cardiologist’s interpretation, exams were audited by the study team, composed of three previously trained investigators. All ECGs of patients with suspected COVID-19 in the study period were eligible for this analysis and stored in a specific database.

### Inclusion criteria

Adult patients (≥ 18 years) of both sexes, seen by health professionals in outpatient units with clinical suspicion or laboratory confirmation of COVID-19, whose clinical data and baseline ECG were transmitted through the RTMG app, were screened. This same set of patients was divided into two study groups and one registry, based exclusively on the treatment informed in the online data collection system, as follows:

• Group 1: Patients submitted, at some point during clinical management, to drug therapy with chloroquine, in schemes recommended by the Brazilian Ministry of Health (18): chloroquine diphosphate D1: 500 mg every 12 h (300 mg of chloroquine base) and D2 to D5: 500 mg every 24 h (300 mg of chloroquine base) or hydroxychloroquine sulfate: D1: 400 mg every 12 h and D2 to D5: 400 mg every 24 h.
• Group 2: Patients under standard/supportive clinical treatment for unspecific respiratory syndrome (flu-like syndrome), without any specific drugs for COVID-19.
• Registry (Group 3): Patients submitted, at some point during clinical management, to drug therapy with ivermectin, antibiotics, antivirals, or other specific drugs proposed for COVID-19 at any recommended doses or chloroquine in dose schemes different from those recommended by the Brazilian Ministry of Health.

### Exclusion criteria

Exclusion criteria were insufficient baseline clinical data entered in the ECG app; failure to transmit an interpretable digital ECG; refusal to sign the electronic informed consent form and to participate in the 3- and 14-day telephone clinical follow-up; impossibility to collect minimally comprehensible information during clinical follow-up, from the patients or relatives/companions.

### Evaluation of outcomes

Study arms: Study groups were continuously identified from data entered in the mobile application (prescription of chloroquine or other specific treatments for COVID-19) and during phone follow-up.

a) Clinical follow-up: Clinical outcomes were systematically assessed on the 3rd (−1 or +2 days) and 14th days (±2 days) after the transmission of the first ECG through standardized phone calls. Contacts were made by trained non-medical professionals at the Telehealth Center or remotely, with at least four attempts per patient, using contact data provided in the mobile app. In case of failure in the 3- and 14-day calls, the patient returned to the study queue for late additional attempts. The link to the electronic informed consent was sent to all patients by short message system (SMS), and clinical follow-up was only initiated after its electronic signature. Throughout the study, messages were sent by SMS and messaging app, with information about the study and reminders about follow-up calls and scheduled ECGs. Considering the small number of patients answering follow-up calls, the study protocol was amended. Clinical outcomes were administratively collected through linkage to national mortality and hospitalization databases: Mortality Information System (Sistema de Informação Sobre Mortalidade—SIM), Influenza Epidemiological Surveillance Information System (Sistema de Informação da Vigilância Epidemiológica da Gripe–SIVEP-Gripe), and COVID-19 Notification System (e-SUS Notifica) (Supplementary material 1). Patient-level data on mortality, hospital admissions, and occurrence of severe acute respiratory syndromes were collected for the whole sample after full-access authorization by the Institutional Review Boards and health authorities, and combined with follow-up data.
b) Electrocardiographic data: All patients with adequate clinical data and at least 1 ECG transmitted to the RTMG system were included in the electrocardiographic study. For the assessment of electrocardiographic outcomes, patients in group 1 (chloroquine) were included only when at least one ECG was performed after drug initiation. For group 2, all registered patients were included. Outcomes were recorded if present in any of the ECGs, at baseline (initiation of treatment, at study entry), or follow-up ECGs.

### Outcomes of interest

The following clinical and electrocardiographic outcomes were measured:

Clinical follow-up: The composite primary clinical outcome consisted of: (a) all-cause hospitalization; (b) admission to intensive care unit (ICU); (c) need for invasive mechanical ventilation; (d) all-cause death. The secondary outcomes were individual components of the primary outcome. For the analysis of outcomes including administrative data, the exportation of the raw database was crosslinked by name, date of birth, mother’s name, and social security number. When necessary, source documents were requested.

Electrocardiographic data: The composite primary electrocardiographic outcome consisted of the occurrence of any new major electrocardiographic abnormalities by the Minnesota coding system in baseline (after treatment initiation) or follow-up (14 days or later) ECGs, confirmed by audit when indicated. When more than 1 ECG was available, abnormalities observed in any of them were considered. The list of major abnormalities considered for the primary outcome is detailed in Supplementary Table 1.

### Statistical analysis

Statistical analysis was performed using the R software version 1.4.1717-3 [The R Foundation for Statistical Computing, Vienna, Austria (19)]. The distribution pattern of the variables was evaluated with the Shapiro–Wilk test. Continuous variables were expressed as mean ± standard deviation (SD) or median and interquartile range (IQR), as appropriate. Categorical variables were expressed as absolute values and percentages. Considering the 7.2% case fatality reported in Italy (20), and a 1.3 hazard ratio for mortality in the chloroquine group (21), a sample of 5,830 patients was needed, with a 1:1 distribution between treatment and control groups and at least 463 events, with 80% power to detect the difference in all-cause death. Comparison between treatment groups (group 1, group 2, group 3) was performed using the analysis of variance (ANOVA) *t*-test for continuous variables with normal distribution and the Kruskal–Wallis test for those with non-normal distribution (Mann–Whitney U test for pairwise comparisons). Categorical variables were compared using the chi-square test, and the Fisher’s exact test for pairwise comparisons.

Multivariable logistic regression was used to examine the association between COVID-19 treatment groups and the primary outcome for each study arm separately. Significant variables (*p* < 0.10) in univariate analyses were included in multivariate models. The models (4) for clinical outcomes, with data from phone follow-up, were adjusted as follows: Model 1 unadjusted, Model 2, adjusted for age and sex; Model 3, adjusted for model 2 variables plus cardiovascular risk factors, collected in a clinical interview (asthma, diabetes, heart disease, hypertension, stroke, heart failure, other lung diseases, kidney disease, overweight/obesity); Model 4, adjusted for variables in model 3 plus clinical variables related to the severity of COVID-19 at presentation (defined as dyspnea and persistent fever) collected during a clinical interview. For clinical outcomes, combining phone contact and administrative databases, model 4 was not applied—as data on disease severity was not available through the ECG app for most patients. The same models (1 to 3) utilized for ECG outcomes were adjusted as follows: Model 1 unadjusted, Model 2, adjusted for age and sex; Model 3, adjusted for model 2 variables plus cardiovascular risk factors, collected through the ECG app (hypertension, diabetes, dyslipidemia, coronary artery disease, previous stroke, smoking, chronic kidney disease, Chagas disease, chronic lung disease). A two-tailed significance level of 0.05 was considered statistically significant.

## Results

During 303 days, a total of 6,957 eligible patients had at least one ECG and clinical data submitted to the online system and were included, with a median age of 49.0 (IQR 38.0–62.0) years, 57% women. Of these, 712 (10.2%) were allocated to group 1 (chloroquine), 3,623 (52.1%) to group 2 (control) and 2,622 (37.7%) to group 3 (other treatments). The baseline demographic and clinical characteristics of the study population are detailed in Table 1. Groups were relatively similar; however, group 1 had a lower proportion of women and a lower prevalence of cardiovascular risk factors: hypertension, diabetes, and dyslipidemia. Groups 1 and 3 had a lower prevalence of Chagas disease (Table 1). In terms of drug therapy, in group 1 100% of the patients used chloroquine compounds at recommended doses, 13.6% corticosteroids, 30.9% antibiotics, and 25.0% antiparasitics; in group 2, 6.0% used corticosteroids and 13.2% antibiotics (at regimens not specific for COVID-19); in group 3, 10.8% used corticosteroids, 25.2% antibiotics, 15.3% antiparasitics, 1.7% chloroquine at non-recommended doses and 40% used other drug classes or associations recommended for COVID-19. No patients received antivirals.

**TABLE 1:** Baseline characteristics and rates of clinical outcomes of all patients included in the study (at least 1 ECG and clinical data entered in the online system), combining phone and administrative follow-up data, and comparison between treatment groups.

Variable	Total^1^ (*N* = 6,957)	Group 1 (Chloroquine)^1^ (*N* = 712)	Group 2 (Control)^1^ (*N* = 3,623)	Group 3 (Registry)^1^ (*N* = 2,622)	*p*-value^2^	*post hoc* ^3^
Sex, male (%)	3,022 (43.4%)	364 (51.1%)	1,476 (40.7%)	1,182 (45.1%)	**<0**.**001**	group 1 > group 3 > group 2 (0.000) (0.001)
Age (years), median (IQR)	49.0 (38.0, 62.0)	48.0 (38.0, 60.0)	49.0 (38.0, 63.0)	50.0 (38.0, 62.0)	0.142	–
Days to latest ECG, median (IQR)	22.0 (7.0, 95.0)	9.0 (4.0, 19.0)	49.0 (11.0, 129.0)	16.0 (7.0, 68.5)	**<0**.**001**	group 1 < group 3 < group 2 (0.000) (0.000)
Hypertension, *N* (%)	3,187 (45.8%)	261 (36.7%)	1,707 (47.1%)	1,219 (46.5%)	**<0**.**001**	group 1 < group 2 = group 3 (0.000) (0.626)
Diabetes, *N* (%)	1,116 (16.0%)	84 (11.8%)	573 (15.8%)	459 (17.5%)	**<0**.**001**	group 1 < group 2 = group 3 (0.001) (0.077)
Dyslipidemia, *N* (%)	421 (6.1%)	22 (3.1%)	258 (7.1%)	141 (5.4%)	**<0**.**001**	group 1 < group 3 < group 2 (0.000) (0.001)
Coronary artery disease, *N* (%)	181 (2.6%)	12 (1.7%)	101 (2.8%)	68 (2.6%)	0.240	–
Previous stroke, *N* (%)	148 (2.1%)	12 (1.7%)	82 (2.3%)	54 (2.1%)	0.592	–
Smoking, *N* (%)	408 (5.9%)	37 (5.2%)	223 (6.2%)	148 (5.6%)	0.507	–
Chronic kidney disease, *N* (%)	58 (0.8%)	4 (0.6%)	28 (0.8%)	26 (1.0%)	0.452	–
Chagas disease, *N* (%)	79 (1.1%)	3 (0.4%)	54 (1.5%)	22 (0.8%)	**0**.**009**	group 1 = group 3 < group 2 (0.221) (0.003)
Chronic lung disease, *N* (%)	63 (0.9%)	7 (1.0%)	32 (0.9%)	24 (0.9%)	0.965	–
**Outcomes**						
Major ECG abnormality, *N* (%)*	1,020 (14.7%)	89 (12.5%)	547 (15.1%)	384 (14.6%)	0.201	–
Major ECG abnormality (follow-up)*	156 (17.0%)	12 (14.8%)	98 (19.5%)	46 (13.8%)	0.082	–
Hospital admission, *N* (%)	1,014 (14.6%)	139 (19.5%)	399 (11.0%)	476 (18.2%)	**<0**.**001**	group 1 = group 3 > group 2 (0.406) (0.000)
ICU admission, *N* (%)	269 (3.9%)	31 (4.4%)	98 (2.7%)	140 (5.3%)	**<0**.**001**	group 1 = group 3 > group 2 (0.281) (0.000)
Mechanical ventilation, *N* (%)	148 (2.1%)	21 (2.9%)	56 (1.5%)	71 (2.7%)	**0**.**002**	group 1 = group 3 < group 2 (0.729) (0.000)
Death, *N* (%)	462 (6.6%)	56 (7.9%)	200 (5.5%)	206 (7.9%)	**<0**.**001**	group 1 = group 3 > group 2 (0.994) (0.000)
Composite clinical outcome, *N* (%)	1,225 (17.6%)	162 (22.8%)	503 (13.9%)	560 (21.4%)	**<0**.**001**	group 1 = group 3 > group 2 (0.425) (0.000)

^1^*n* (%); median (IQR). ^2^Pearson’s Chi-squared test; Kruskal–Wallis rank sum test. ^3^Fisher’s exact test. *For ECGs obtained at baseline and/or follow-up. Number of patients with late follow-up ECG: 917 (13.2%) patients [group 1: 81 (11.4%), group 2: 512 (14.1%), group 3: 334 (12.7%), *p* = 0.004]. Bold values represent a *p*-value < 0.05.

Throughout the study period, there was a decreasing trend in daily entries, with no significant increase during the 2nd peak of the COVID-19 pandemic in Brazil (April to June 2021) (Supplementary Figure 1). A late (≥ 14 days) follow-up ECG was obtained for 917 (13.2%) patients [group 1: 81 (11.4%), group 2: 512 (14.1%), group 3: 334 (12.7%)].

Regarding individual components of the primary outcome, crude rates of hospitalization, ICU admission, mechanical ventilation and all-cause death were higher in groups 1 (chloroquine) and 3 (other treatments) compared to group 2 (control). A total of 462 deaths were recorded. Rates of the primary ECG endpoint, however, were similar (Table 1).

### Clinical outcomes–phone follow-up

At total, 1,969 (28.3%) patients responded the clinical phone follow-up: 260 (13.2%) patients in group 1, 871 (44.2%) in group 2 and 838 (42.6%) in group 3. Baseline demographic and clinical characteristics of this subpopulation, as well as COVID-19 symptoms at presentation, are detailed in Table 2. The groups were overall similar, except for the significantly higher proportion of women in group 2, younger age and lower prevalence of hypertension in group 1, lower prevalence of lung diseases in groups 1 and 2, and higher incidence of severe COVID-19 symptoms (fever and dyspnea) in groups 1 and 3, compared to controls (group 2) (Table 2).

**TABLE 2:** Baseline characteristics and rates of outcomes of interest of patients included in the phone clinical follow-up, and comparison between treatment groups.

Variable	Total^1^ (*N* = 1,969*)	Group 1 (Chloroquine)^1^ (*N* = 260)	Group 2 (Control)^1^ (*N* = 871)	Group 3 (Registry)^1^ (*N* = 838)	*p*-value^2^	*post hoc* ^3^
Sex, male (%)	869 (44.1%)	135 (51.9%)	353 (40.5%)	381 (45.5%)	**0**.**003**	group 2 > group 1 = group 3 (0.004) (0.069)
Age (years), median (IQR)	47.0 (37.0, 59.0)	45.0 (35.0, 55.0)	47.0 (37.0, 60.0)	47.0 (37.0, 59.8)	**0**.**022**	group 1 < group 2 (0.046) group 1 < group 3 (0.020)
Asthma, *N* (%)	55 (2.8%)	3 (1.2%)	23 (2.6%)	29 (3.5%)	0.134	–
Diabetes, *N* (%)	269 (13.7%)	31 (11.9%)	115 (13.2%)	123 (14.7%)	0.460	–
Cardiac disease, *N* (%)	136 (6.9%)	19 (7.3%)	55 (6.3%)	62 (7.4%)	0.652	–
Hypertension, *N* (%)	770 (39.1%)	77 (29.6%)	338 (38.8%)	355 (42.4%)	**0**.**001**	group 1 < group 2 = group 3 (0.001) (0.134)
Myocardial infarction, *N* (%)	23 (1.2%)	0 (0.0%)	11 (1.3%)	12 (1.4%)	0.125	–
Heart failure, *N* (%)	5 (0.3%)	0 (0.0%)	2 (0.2%)	3 (0.4%)	0.843	–
Other lung diseases, *N* (%)	60 (3.0%)	4 (1.5%)	20 (2.3%)	36 (4.3%)	**0**.**018**	group 1 = group 2 < group 3 (0.441) (0.006)
Overweight/obesity, *N* (%)	286 (14.5%)	44 (16.9%)	117 (13.4%)	125 (14.9%)	0.342	–
Kidney failure and/or dialysis, *N* (%)	10 (0.5%)	0 (0.0%)	6 (0.7%)	4 (0.5%)	0.457	–
Other kidney diseases, *N* (%)	34 (1.7%)	4 (1.5%)	11 (1.3%)	19 (2.3%)	0.279	–
Shortness of breath, *N* (%)	820 (41.6%)	117 (45.0%)	319 (36.6%)	384 (45.8%)	**<0**.**001**	group 2 < group 1 = group 3 (0.000) (0.816)
Fever, *N* (%)	968 (49.2%)	132 (50.8%)	390 (44.8%)	446 (53.2%)	**0**.**002**	group 2 < group 1 = group 3 (0.001) (0.489)
Prostration, *N* (%)	745 (37.8%)	103 (39.6%)	326 (37.4%)	316 (37.7%)	0.812	
**Outcomes**						
Hospital admission, *N* (%)	544 (27.6%)	100 (38.5%)	157 (18.0%)	287 (34.2%)	**<0**.**001**	group 2 < group 1 = group 3 (0.000) (0.216)
ICU admission, *N* (%)	94 (4.8%)	8 (3.1%)	27 (3.1%)	59 (7.0%)	**<0**.**001**	group 3 > group 1 = group 2 (0.000) (0.985)
Mechanical ventilation, *N* (%)	33 (1.7%)	3 (1.2%)	14 (1.6%)	16 (1.9%)	0.758	–
Death, *N* (%)	24 (1.2%)	3 (1.2%)	8 (0.9%)	13 (1.6%)	0.533	–
Composite clinical outcome, *N* (%)	544 (27.6%)	100 (38.5%)	157 (18.0%)	287 (34.2%)	**<0**.**001**	group 2 < group 1 = group 3 (0.000) (0.216)

^1^*n* (%); median (IQR). ^2^Pearson’s Chi-squared test; Kruskal–Wallis rank sum test. ^3^Fisher’s exact test. *Patients who answered the phone follow-up. Bold values represent a *p*-value < 0.05.

Among individual clinical outcomes, higher hospitalization rates were observed in groups 1 (38.5%) and 3 (34.2%) compared to the control group (2) (18.0%), *p* < 0.001, in addition to higher ICU admission rates in group 3 (7.0%) compared to groups 1 (3.1%) and 2 (3.1%), *p* < 0.001. Mechanical ventilation and death rates were similar (Table 2). The composite primary endpoint occurred more frequently in groups 1 (38.5%) and 3 (34.2%) compared to group (2) (18.0%), *p* < 0.001 (Table 2).

In logistic regression models, prescription of chloroquine was independently associated with a 2.8-fold greater chance of the primary composite outcome, compared with the control group, in the unadjusted model [OR: 2.84 (95% CI 2.10–3.85), *p* < 0.001], as well as in models: 2 [adjusted for sex and age; OR: 3.17 (95% CI 2.31–4.35), *p* < 0.001]; 3 [adjusted for sex, age and risk factors; OR: 3.23 (95% CI 2.34–4.45), *p* < 0.001] and 4 [adjusted for sex, age, risk factors and variables of COVID-19 presentation; OR: 3.24 (95% CI 2.31–4.54), *p* < 0.001]. A similar association was observed for group 3, which also associated with a 2.4-fold greater risk of occurrence of the primary outcome in the unadjusted model 1 [OR: 2.37 (95% CI 1.90–2.97), *p* < 0.001], as well as in the adjusted models (2, 3, and 4) (Table 3).

**TABLE 3:** Multivariate risk model for the composite primary outcome (hospitalization/ICU admission/mechanical ventilation/death) assessed by phone follow-up, adjusted for demographic, clinical, and COVID-19 presentation-related variables.

Variables (*N* = 1,969)	OR (95% CI)	*P*-value
**Model 1**		
(Intercept)	0.22 (0.18–0.26)	**<0**.**001**
Group 1 (chloroquine)	2.84 (2.10–3.85)	**<0**.**001**
Group 3 (registry: other treatments)	2.37 (1.90–2.97)	**<0**.**001**
**Model 2**		
(Intercept)	0.03 (0.02–0.05)	**<0**.**001**
Group 1 (chloroquine)	3.17 (2.31–4.35)	**<0**.**001**
Group 3 (registry: other treatments)	2.40 (1.91–3.04)	**<0**.**001**
Sex (Male)	1.78 (1.45–2.20)	**<0**.**001**
Age	1.03 (1.03–1.04)	**<0**.**001**
**Model 3**		
(Intercept)	0.03 (0.02–0.05)	**<0**.**001**
Sex (Male)	1.88 (1.52–2.33)	**<0**.**001**
Age	1.03 (1.02–1.04)	**<0**.**001**
Group 1 (chloroquine)	3.23 (2.34–4.45)	**<0**.**001**
Group 3 (registry: other treatments)	2.37 (1.88–3.01)	**<0**.**001**
Asthma	0.82 (0.40–1.59)	0.572
Diabetes	2.00 (1.49–2.69)	**<0**.**001**
Cardiac disease	1.10 (0.73–1.63)	0.651
Hypertension	1.06 (0.83–1.34)	0.646
Myocardial infarction	1.14 (0.46–2.76)	0.776
Heart failure	3.62 (0.52–30.44)	0.193
Other lung diseases	2.15 (1.22–3.76)	**0**.**008**
Kidney disease and/or dialysis	1.22 (0.24–4.8)	0.788
Overweight/Obesity	1.57 (1.16–2.09)	**0**.**003**
**Model 4**		
Sex (Male)	1.81 (1.44–2.27)	**<0**.**001**
Age	1.03 (1.03–1.04)	**<0**.**001**
Group 1 (chloroquine)	3.24 (2.31–4.54)	**<0**.**001**
Group 3 (registry: other treatments)	2.21 (1.73–2.85)	**<0**.**001**
Asthma	0.64 (0.30–1.28)	0.217
Diabetes	1.85 (1.36–2.53)	**<0**.**001**
Cardiac disease	1.02 (0.66–1.55)	0.933
Hypertension	1.03 (0.80–1.32)	0.837
Heart failure	4.24 (0.57–35.32)	0.144
Other lung diseases	1.7 (0.94–3.07)	0.076
Kidney disease and/or dialysis	1.00 (0.18–4.60)	0.998
Overweight/Obesity	1.27 (0.93–1.73)	0.131
Shortness of breath	3.75 (2.99–4.73)	**<0**.**001**
Fever	2.03 (1.61–2.55)	**<0**.**001**

Bold values represent a *p*-value < 0.05.

### Clinical outcomes—phone follow-up plus national administrative databases

When outcome data from phone follow-up and administrative databases were combined (*N* = 6,957), the composite primary endpoint also occurred more frequently in groups 1 (22.8%) and 3 (21.4%) compared to controls (2) (13.9%), *p* < 0.001 (Table 1). Chloroquine was again associated with a 1.8-fold greater chance of the primary outcome compared to the control group (2), in the unadjusted model [OR: 1.83 (95% CI 1.49–2.23), *p* < 0.001], with similar effects observed in models 2 [adjusted for sex and age; OR: 1.96 (95% CI 1.59–2.41), *p* < 0.001] and 3 [adjusted for sex, age and risk factors; OR: 1.99 (95% CI 1.61–2.44), *p* < 0.001]. Group 3 was also independently associated with the primary outcome, although with a lesser magnitude (Table 4).

**TABLE 4:** Multivariate risk model for the composite primary outcome (hospitalization/ICU/mechanical ventilation/death) assessed by phone follow-up plus administrative databases, and for the primary electrocardiographic outcome (major ECG abnormalities by the Minnesota code), adjusted for demographic and clinical variables.

Variable (*N* = 6,957)	OR (95% CI)	*P*-value	OR (95% CI)	*P*-value
**Outcome**	**Hospitalization/ICU admission/mechanical ventilation/death**		Major ECG abnormalities*	
**Model 1**				
(Intercept)	0.16 (0.15–0.18)	**<0**.**001**	0.18 (0.16–0.19)	**<0**.**001**
Group 1 (chloroquine)	1.83 (1.49–2.23)	**<0**.**001**	0.80 (0.63–1.02)	0.074
Group 3 (registry: other treatments)	1.68 (1.48–1.92)	**<0**.**001**	0.96 (0.84–1.11)	0.620
**Model 2**				
(Intercept)	0.02 (0.01–0.02)	**<0**.**001**	0 (0–0.01)	**<0**.**001**
Group 1 (chloroquine)	1.96 (1.59–2.41)	**<0**.**001**	0.85 (0.65–1.09)	0.203
Group 3 (registry: other treatments)	1.72 (1.50–1.97)	**<0**.**001**	0.93 (0.80–1.09)	0.389
Sex (Male)	1.51 (1.33–1.72)	**<0**.**001**	1.82 (1.58–2.11)	**<0**.**001**
Age	1.04 (1.03–1.04)	**<0**.**001**	1.06 (1.06–1.07)	**<0**.**001**
**Model 3**				
(Intercept)	0.02 (0.01–0.02)	**<0**.**001**	0 (0–0.01)	**<0**.**001**
Sex (Male)	1.52 (1.34–1.73)	**<0**.**001**	1.9 (1.64–2.21)	**<0**.**001**
Age	1.04 (1.03–1.04)	**<0**.**001**	1.06 (1.05–1.06)	**<0**.**001**
Group 1 (chloroquine)	1.99 (1.61–2.44)	**<0**.**001**	0.92 (0.71–1.19)	0.542
Group 3 (registry: other treatments)	1.70 (1.48–1.96)	**<0**.**001**	0.97 (0.83–1.13)	0.704
Hypertension	1.00 (0.86–1.15)	0.965	1.48 (1.25–1.74)	**<0**.**001**
Diabetes	1.38 (1.17–1.63)	**<0**.**001**	0.95 (0.79–1.14)	0.607
Dyslipidemia	0.88 (0.68–1.14)	0.351	1.18 (0.91–1.52)	0.214
Coronary artery disease	1.04 (0.71–1.50)	0.844	3.13 (2.23–4.38)	**<0**.**001**
Previous stroke	1.39 (0.93–2.05)	0.102	1.05 (0.69–1.57)	0.819
Smoking	0.84 (0.64–1.10)	0.223	0.95 (0.70–1.26)	0.713
Chronic kidney disease	1.33 (0.70–2.42)	0.364	1.04 (0.49–2.06)	0.924
Chagas disease	1.27 (0.72–2.13)	0.391	3.76 (2.29–6.14)	**<0**.**001**
Chronic lung disease	1.21 (0.64–2.16)	0.544	0.76 (0.38–1.46)	0.429

ECG: electrocardiogram; ICU: intensive care unit. *For ECGs obtained at baseline and/or follow-up. Number of patients with late follow-up ECG: 917 (13.2%) patients (group 1: 81 (11.4%), group 2: 512 (14.1%), group 3: 334 (12.7%), *p* = 0.004). Bold values represent a *p*-value < 0.05.

In the analysis of secondary outcomes, chloroquine was also independently associated with higher all-cause mortality, with a 1.7-fold increase in the final adjusted model (3) (Supplementary Table 2).

### Electrocardiographic outcomes

The detailed comparison between electrocardiographic characteristics between groups, at baseline and follow-up, is depicted in Supplementary Table 2. The occurrence of the primary ECG outcome (*N* = 6,957) was similar between groups (group 1: 12.5%; group 2: 15.1%; group 3: 14.6%, *p* = 0.201). In multivariable logistic models, the use of chloroquine was not associated with a higher occurrence of the primary ECG endpoint in the unadjusted model, nor in the models adjusted for sex, age and risk factors (Table 4). Likewise, the prescription of other specific treatments for COVID-19 (group 3) was also not associated with the occurrence of the primary ECG endpoint during COVID-19 treatment (Table 4).

## Discussion

Our study, with a large sample of outpatients with clinical suspicion of COVID-19 in different regions of Brazil, showed that the prescription of chloroquine did not increase major ECG abnormalities compared to patients without specific treatment. On the other hand, chloroquine was associated with higher rates of adverse outcomes, noticeably hospitalization. This effect was consistent after adjustments for multiple variables, including clinical comorbidities and severity of COVID-19 at presentation.

Since the inception of the COVID-19 pandemic, given the great transmissibility and high mortality of the disease—especially in individuals with high cardiovascular risk—there has been great interest in the investigation of specific treatments for early and advanced stages aimed at the control of the exaggerated inflammatory response. In this context, chloroquine emerged as a potential option, and, despite the absence of robust evidence on its effectiveness and safety, there were recommendations for its widespread use in several countries (22). Chloroquine belongs to the class of quinoline antimalarials and blocks the fast-activating delayed rectifier potassium current (23), coded by the human ether-related gene (hERG), in a concentration and time-dependent manner. Such inhibition of the hERG K+ channel can lead to prolongation of the action potential duration and, consequently, of the QT interval on the ECG, potentially triggering ventricular arrhythmias (24). The cardiovascular risk is theoretically further potentialized by the higher incidence of cardiac arrhythmias and acute myocardial injury including myocarditis—a pro-arrhythmogenic condition (25, 26)—in severe COVID-19 phenotypes, markedly in individuals requiring intensive care. Therefore, drugs that prolong the QTc can presumably exacerbate the risk of underlying arrhythmia (27).

Doubts about the real benefits of chloroquine in COVID-19, associated with its potential risks and the absence of other treatments with an impact on mortality, prompted the design of several clinical studies involving thousands of patients from different continents in a variety of disease presentations and stages. There was particular interest in the so-called “early treatment,” with the hypothesis that chloroquine, administered after contact with a confirmed case or in the early stages of the flu-like syndrome, could prevent progression to severe forms. One of the most extensive studies, with 2,314 contacts of COVID-19 patients randomized between hydroxychloroquine (its less toxic hydroxylated metabolite) and usual care, demonstrated similar rates of symptomatic COVID-19 (hydroxychloroquine 5.7% vs. placebo 6.2%, *p* = NS) and disease transmission (18.7 vs. 17.8%, *p* = NS), with non-severe side effects reported in the treatment arm (28). Another large-scale randomized trial showed similar results (COVID-19 incidence 11.8 vs. 14.3%, *p* = NS), with higher rates of adverse effects in the hydroxychloroquine group (29). Similarly, hydroxychloroquine also did not result in less positive diagnostic tests at 28 days in an open study including 150 patients, again with higher rates of side effects in those receiving the drug, including two serious events (30). In none of these studies adverse electrocardiographic outcomes were reported in subjects using chloroquine, although this was not the outcome of interest.

Our sample reflects the context of outpatient treatment of COVID-19, predominantly at early stages and in primary care or emergency public units. Similar to the findings of most primary studies, we observed no clinical benefit of chloroquine; conversely, the drug was independently associated with higher rates of the primary composite outcome, especially at the expense of higher hospitalization rates [adjusted OR: 3.24 (95% CI 2.31–4.54)]. The findings were reinforced by the combination of phone follow-up and administrative data for outcome assessment. However, this trend should be cautiously analyzed, considering the study’s methodology (observational, non-randomized, and with secondary data) and the enrollment strategy, based on clinical suspicion of COVID-19—not necessarily confirmed—often raised in units with limited technical resources. However, a meta-analysis of 28 randomized and non-randomized studies published until October 2020, involving over 13,000 patients with COVID-19, showed comparable results, with increased mortality (consistent across all sensitivity analyses) with hydroxychloroquine (OR: 1.11, 95% CI 1.02–1.20, *I*^2^ = 0%) and a similar trend, with smaller sample size, for chloroquine (OR: 1.77, 95% CI 0.15–21.13, *I*^2^ = 0%) (31). Among other factors, this effect may be associated with the dose regimens of chloroquine, especially at the beginning of the pandemic, since interim analyzes of clinical trials showed an increase in both ECG abnormalities (especially prolonged QTc) and lethality with the prescription of higher doses (32). In our protocol, the chloroquine group consisted of patients using low doses standardized by the Brazilian Ministry of Health (18). However, inaccuracies in filling data in the ECG app and difficulties inherent to secondary—and frequently retrospective—data collection through phone contact may have led to the inclusion of different and potentially risky therapeutic regimens (32). It may also be hypothesized that the prescription of chloroquine as a presumable effective treatment may have delayed or deferred the access to guideline-driven supportive therapies or even to hospital and intensive care.

In trials in other clinical scenarios involving patients in late and more severe stages of COVID-19, including those in the ICU, chloroquine also did not improve clinical status during hospitalization, nor did they have an impact on mortality (33), with a trend toward clinical deterioration in some studies (34). Likewise, different studies failed to demonstrate any benefit of combining such compounds with other specific treatments—including antibiotics such as azithromycin, antiparasitics such as ivermectin, and antivirals—in moderate to severe COVID-19 (12, 13, 35). Although clinical benefit was null, no robust data suggest an increase in serious adverse effects and unfavorable outcomes with these drugs at their usual doses. This also contrasts, in a way, with our results, since the use of other specific COVID-19 treatments—excluding recommended chloroquine doses—(parallel registry) was also associated with worse outcomes [adjusted OR: 2.21 (95% CI 1.73–2.85)]. Again, this finding should be carefully interpreted in light of the methodology applied and—especially for the registry—considering the heterogeneity of the treatments in terms of drug classes, doses, and associations. Despite the consistency of the effect after multiple adjustments, biases resulting from such a degree of heterogeneity may have impacted the results.

Regarding ECG data, the lack of association between chloroquine prescription and major ECG abnormalities or arrhythmias was consistent across different model adjustments, and no association was observed between other treatments (registry) and the primary ECG outcome. Despite the challenges for the acquisition of longitudinal electrocardiographic data in a nationwide study carried out by a public telemedicine network, this finding is in agreement with most studies published so far (36). Despite the potential risk of cardiac arrhythmias associated with antimalarial drugs, in addition to the cardiac involvement in severe COVID-19 and the preliminary case reports of potentially fatal arrhythmias in patients with the disease (37), studies with chloroquine in both hospital and outpatient settings have not consistently shown an increase in major ECG changes, despite the myriad of incident side effects in the treatment groups (36, 38). Data heterogeneity should also be considered for this analysis, especially about the timing of the baseline ECG versus the peak incidence of electrocardiographic outcomes following chloroquine administration (first 72 h) and the considerable loss of electrocardiographic follow-up. As the majority of ECGs analyzed preceded the peak of the drug’s arrhythmogenic effect, with very limited 3-day and 14-day tracings, definite causal inferences cannot be drawn from our study. On the other hand, there are no consistent evidences of late electrocardiographic effects of chloroquine, especially after discontinuation. Furthermore, major ECG abnormalities were associated with variables as age, gender, hypertension, coronary artery disease and Chagas’ disease—all known risk factors for cardiac pathologies. Thus, they may possibly be due to underlying cardiovascular disease, and not directly related to COVID-19 or chloroquine.

Even with the aforementioned difficulties for the acquisition of longitudinal data, tele-ECG emerged as a promising tool to support risk stratification and decision-making during a pandemic. Although the strategy may not be feasible in certain areas—noticeably remote locations with limited connection—worldwide studies suggest that tele-ECG may help guide effective control and interventions, including from low- and middle-income countries where documentation of cardiovascular abnormalities and risk factors in COVID-19 patients is scarce (39). Furthermore, other technology-based solutions as wearable devices and smartwatches hold promise for individuals with respiratory diseases. They have been successfully tested to predict the onset of COVID-19 through early changes in heart rate variability, to track the effects of vaccination on the body and to monitor normalization of heart rate after SARS-CoV-2 infection, as a surrogate for long COVID-19 (40, 41). This opens up a route for clinical application of biometric data.

Despite the challenges of conducting large-scale population-based research, with clinical and ECG data collection in a continental country, our study represents the largest Latin American outpatient sample with real-life data. Although outcomes should be parsimoniously analyzed, mainly due to the high rate of loss-to-follow-up—requiring cross-linkage with administrative databases—and the possibility of selection (greater response to phone contacts in families who experienced severe cases, with closer connection with health services) and treatment (drugs most often administered to severe cases) biases, the final models are consistent. Even after detailed adjustment for demographic and clinical variables, comorbidities, and COVID-19 symptoms at presentation, the association between chloroquine and unfavorable outcomes—especially hospitalization—remained broadly significant, with OR > 3.0. Furthermore, the effect was strengthened by the inclusion of administrative data, suggesting that these findings should be considered for therapeutic decision-making. On the other hand, robust ECG data suggest the safety of chloroquine and other drug regimens in terms of the induction of rhythm disturbances and major abnormalities, even with the increased risk associated with the potential severity of the disease, inflammatory response, cardiac involvement, and coexistence of cardiovascular disease. In the absence of ECG changes explaining the worse outcomes among treated patients, it may be hypothesized that late incident arrhythmias, or other severe side effects may have accounted for higher rates of clinical events. Thus, continuous efforts should be made to mitigate the risks of cardiac and systemic toxicity (42).

### Limitations

Our study has several limitations, which should be considered to interpret the findings. First, data collection was performed indirectly, through telephone contact, information entered into the ECG app by the provider, or cross-linkage with national mortality and hospitalization databases. Despite the pragmatic research protocol, there may have been some imprecision in data collection, especially regarding details and timing of outcomes. Markedly for the phone contact arm, there is potential bias related to the precision of outcome assessment as well as to misinterpretation of clinical questions by patients and families. Patient literacy—not systematically evaluated in the study—may have contributed to this issue. Second, there was a great difficulty in the completion of telephone follow-up, underpowering this specific analysis and possibly selecting individuals with access to communication and mobile devices and better acceptance of the approach by the research team. The heterogeneous timing of the late phone follow-up, noticeably when several attempts were needed, may have also affected outcome rates. Although this sample with detailed clinical information was limited, the magnitude of the observed effects was robust and maintained despite several adjustments. Third, the rates of ECG follow-up were extremely low. Although treatment-related ECG abnormalities usually develop early (being detectable in the 3-day ECG), our data do not allow for longitudinal analyses of the incidence of ECG changes during COVID-19. Fourth, the inclusion criteria were broad, and patients were enrolled regardless of laboratory diagnosis of COVID-19. As COVID-19 tests were not broadly available in Brazil in the beginning of the pandemic, data on positivity and type of test was not available. Finally, detailed information about causes of hospitalization and death was not possible by remote contact nor by the non-qualified databases, limiting inferences about underlying and associated conditions. Despite these limitations, to the best of our knowledge, this is the largest study with clinical and electrocardiographic follow-up of outpatients with COVID-19 in Latin America. The results are representative of real-life patient care during the pandemic. Added to available data, these findings may help consolidate evidence-based recommendations for the treatment of COVID-19.

## Conclusion

Chloroquine was associated with a higher risk of poor outcomes in patients suspected to have COVID-19 when compared to those who received standard care. The utilization of other specific treatments for COVID-19 in the parallel registry was also associated with an equally higher risk. Follow-up ECGs were obtained in only 13.2% of patients and did not show any significant differences in major abnormalities amongst the three groups. In the absence of early incident ECG abnormalities, other side effects of chloroquine, late arrhythmias or deferral of medical care may explain the worse outcomes.

Such data add to the evidence on the non-efficacy and potential risk of treating COVID-19 with chloroquine. However, limitations inherent to the observational study design and the remote and indirect collection of non-randomized data preclude definite causal inferences.

## Data availability statement

The raw data supporting the conclusions of this article will be made available by the authors, without undue reservation.

## Ethics statement

The studies involving human participants were reviewed and approved by the Institutional Review Board of Universidade Federal de Minas Gerais, CAAE number 37228120.9.0000.5149. The patients/participants provided their written informed consent to participate in this study.

## Author contributions

BN, AR, and GP: conception and design of the research. LT, AA, DP, LR, PG, and MM: acquisition of data. AR, BN, and GP: analysis and interpretation of data. MP and BN: statistical analysis. BN, GP, and AR: writing of the manuscript. BN, AR, and GP: responsible for the overall content as guarantors. All authors contributed critical revision of the manuscript for intellectual content and approved the submitted version.
