# Supplementary material for: Clinical and electrocardiographic outcomes evaluated by telemedicine of outpatients with clinical suspicion of COVID-19 treated with chloroquine compounds in Brazil†
Source: Front Cardiovasc Med. 2023 Feb 15;10:1028398. doi: 10.3389/fcvm.2023.1028398 (PMC9978955; doi:10.3389/fcvm.2023.1028398)
Supplement: Supplementary file 2 [file Table_2.docx]

**Table S1:** Major abnormalities, based on the Minnesota criteria, considered for the primary electrocardiographic endpoint.

| **Type of variable:** | **Major abnormality:** |
| --- | --- |
| **Heart rate** | Sinus bradycardia, sinus tachycardia |
| **Supraventricular arrhythmias** | Ectopic atrial rhythm , multifocal atrial rhythm, atrial tachycardia, multifocal atrial tachycardia, paroxysmal supraventricular tachycardia, junctional rhythm, atrial fibrillation and flutter, supraventricular premature beats |
| **Ventricular arrhythmias** | Ventricular premature beats, non-sustained ventricular tachycardia, sustained ventricular tachycardia, accelerated idioventricular rhythm |
| **Atrioventricular conduction disturbances** | Atrioventricular block: 1st degree , 2^nd^ degree ( Mobitz 1 and 2), 2:1, advanced and total; ventricular pre-excitation |
| **Intraventricular conduction disturbances** | Right bundle-branch block, left bundle-branch block, right bundle-branch block + left anterosuperior divisional block, right branch + posteroinferior divisional block and left, intraventricular block |
| **Myocardial ischemia** | ST segment: supra (subepicardial injury), supra-ST (subepicardial injury, probable acute myocardial infarction), infra (subendocardial injury), infra-ST , T POS (subendocardial ischemia), electrically inactive area |
| **Chamber overload** | Left ventricular overload (Romhilt Estes criterion) |
| **Implantable devices** | Pacemaker rhythm (normal); pacemaker rhythm (abnormal) |
| **Other repolarization abnormalities** | Prolonged QT interval, primary changes in ventricular repolarization |

**Table S2:** Multivariate risk model for the all-cause mortality, assessed by phone follow-up plus administrative databases, adjusted for demographic and clinical variables.

| **Variable (N = 6,957):** | **OR (95% CI)** | **P-value** |
| --- | --- | --- |
| **Model 1:** |  |  |
| **(Intercept)** | 0.06 (0.05-0.07) | **< 0.001** |
| ***Group 1 (Chloroquine)*** | 1.46 (1.07-1.97) | 0.016 |
| ***Group 3 (Registry: other treatments)*** | 1.46 (1.19-1.79) | **< 0.001** |
| **Model 2:** |  |  |
| **(Intercept)** | 0 (0-0) | **< 0.001** |
| ***Group 1 (Chloroquine)*** | 1.65 (1.19-2.25) | **0.002** |
| ***Group 3 (Registry: other treatments)*** | 1.48 (1.21-1.82) | **< 0.001** |
| **Sex (Male)** | 1.09 (0.89-1.32) | 0.403 |
| **Age** | 1.05 (1.05-1.06) | **< 0.001** |
| **Model 3:** |  |  |
| **(Intercept)** | 0 (0-0) | **< 0.001** |
| **Sex (Male)** | 1.09 (0.89-1.33) | 0.392 |
| **Age** | 1.05 (1.04-1.06) | **< 0.001** |
| ***Group 1 (Chloroquine)*** | 1.67 (1.2-2.28) | **0.002** |
| ***Group 3 (Registry: other treatments)*** | 1.48 (1.2-1.82) | **< 0.001** |
| **Hypertension** | 1.04 (0.84-1.3) | 0.705 |
| **Diabetes** | 1.19 (0.93-1.5) | 0.161 |
| **Dyslipidemia** | 0.9 (0.62-1.29) | 0.592 |
| **Coronary artery disease** | 1.16 (0.67-1.91) | 0.567 |
| **Previous stroke** | 0.99 (0.53-1.71) | 0.971 |
| **Smoking** | 1.05 (0.7-1.53) | 0.789 |
| **Chronic kidney disease** | 0.7 (0.21-1.78) | 0.502 |
| **Chagas disease** | 1.12 (0.46-2.33) | 0.786 |
| **Chronic lung disease** | 0.84 (0.28-1.97) | 0.71 |

* P<0.05.

**Table S3:** Baseline and follow-up echocardiographic major abnormalities, QT interval and QT index (>116 milliseconds) of all patients included in the study, rates of progression (development) of each abnormality and comparison between groups.

| **Minnesota Category*:** | **Overall**  **N = 6,957*^1^*** | **Group 1 (Chloroquine) *^1^***  **N = 712*^1^*** | **Group 2 (Control) *^1^***  **N = 3,623** | **Group 3 (Registry) *^1^***  **N = 2,622** | **p-value*^2^*** | **post hoc *^3^*** |
| --- | --- | --- | --- | --- | --- | --- |
| **Baseline** |  |  |  |  |  |  |
| **m1** | 202 (2.9%) | 23 (3.2%) | 96 (2.6%) | 83 (3.2%) | 0.420 |  |
| **m2** | 305 (4.4%) | 41 (5.8%) | 142 (3.9%) | 122 (4.7%) | 0.063 |  |
| **m3** | 283 (4.1%) | 18 (2.5%) | 167 (4.6%) | 98 (3.7%) | **0.020** | group 1 < group 2 = group 3 (0.019)(0.090) |
| **m4** | 172 (2.5%) | 23 (3.2%) | 77 (2.1%) | 72 (2.7%) | 0.115 |  |
| **m5** | 341 (4.9%) | 45 (6.3%) | 141 (3.9%) | 155 (5.9%) | **<0.001** | group 1 = group 3 > group 2  (0.686)(0.000) |
| **m6** | 393 (5.6%) | 39 (5.5%) | 203 (5.6%) | 151 (5.8%) | 0.945 |  |
| **m7** | 661 (9.5%) | 63 (8.8%) | 332 (9.2%) | 266 (10.1%) | 0.351 |  |
| **m8** | 1,352 (19.4%) | 143 (20.1%) | 683 (18.9%) | 526 (20.1%) | 0.442 |  |
| **m9** | 937 (13.5%) | 117 (16.4%) | 453 (12.5%) | 367 (14.0%) | **0.012** | group 1 > group 2 = group 3  (0.106)(0.017) |
| **QTc (ms, median, IQR)** | 418.0 (404.0, 433.0) | 415.0 (402.0, 429.0) | 418.0 (405.0, 433.0) | 417.0 (404.0, 432.0) | **<0.001** | group 1 < group 2 = group 3  (0.010)(0.176) |
| **iQT>=116 ms** | 6,929 (99.6%) | 711 (99.9%) | 3,605 (99.5%) | 2,613 (99.7%) | 0.410 |  |
| **Minnesota Category:** | **Overall**  **N = 917*^1^*** | **Group 1 (Chloroquine) *^1^***  **N = 712*^1^*** | **Group 2 (Control) *^1^***  **N = 3,623** | **Group 3 (Registry) *^1^***  **N = 2,622** | **p-value***^2^* | **post hoc** *^3^* |
| **Follow-up:** |  |  |  |  |  |  |
| **m1** | 46 (5.0%) | 2 (2.5%) | 20 (4.0%) | 24 (7.2%) | 0.080 |  |
| **m2** | 54 (5.9%) | 3 (3.7%) | 32 (6.4%) | 19 (5.7%) | 0.626 |  |
| **m3** | 61 (6.7%) | 2 (2.5%) | 37 (7.4%) | 22 (6.6%) | 0.259 |  |
| **m4** | 38 (4.1%) | 2 (2.5%) | 18 (3.6%) | 18 (5.4%) | 0.349 |  |
| **m5** | 78 (8.5%) | 5 (6.2%) | 39 (7.8%) | 34 (10.2%) | 0.347 |  |
| **m6** | 89 (9.7%) | 8 (9.9%) | 51 (10.2%) | 30 (9.0%) | 0.852 |  |
| **m7** | 122 (13.3%) | 10 (12.3%) | 68 (13.5%) | 44 (13.2%) | 0.954 |  |
| **m8** | 196 (21.4%) | 21 (25.9%) | 111 (22.1%) | 64 (19.2%) | 0.344 |  |
| **m9** | 190 (20.7%) | 16 (19.8%) | 88 (17.5%) | 86 (25.7%) | **0.016** | group 1 = group 2 < group 3 (0.632)(0.002) |
| **QTc (ms, median, IQR)** | 421.0 (407.0, 436.0) | 417.0 (403.0, 430.0) | 422.0 (407.0, 437.5) | 419.5 (407.0, 435.8) | 0.147 |  |
| **iQT>=116 ms** | 915 (99.8%) | 81 (100.0%) | 501 (99.8%) | 333 (99.7%) | >0.999 |  |
| **Progression:** |  |  |  |  |  |  |
| **Prog. m1** | 17 (1.9%) | 0 (0.0%) | 7 (1.4%) | 10 (3.0%) | 0.134 |  |
| **Prog. m2** | 29 (3.2%) | 1 (1.2%) | 17 (3.4%) | 11 (3.3%) | 0.714 |  |
| **Prog. m3** | 30 (3.3%) | 1 (1.2%) | 16 (3.2%) | 13 (3.9%) | 0.566 |  |
| **Prog. m4** | 19 (2.1%) | 0 (0.0%) | 10 (2.0%) | 9 (2.7%) | 0.386 |  |
| **Prog. m5** | 37 (4.0%) | 0 (0.0%) | 21 (4.2%) | 16 (4.8%) | 0.112 |  |
| **Prog. m6** | 54 (5.9%) | 6 (7.4%) | 29 (5.8%) | 19 (5.7%) | 0.830 |  |
| **Prog. m7** | 42 (4.6%) | 2 (2.5%) | 25 (5.0%) | 15 (4.5%) | 0.745 |  |
| **Prog. m8** | 99 (10.8%) | 6 (7.4%) | 54 (10.8%) | 39 (11.7%) | 0.539 |  |
| **Prog. m9** | 111 (12.1%) | 8 (9.9%) | 52 (10.4%) | 51 (15.3%) | 0.084 |  |

*^1^* n (%); Median (IQR); *^2^* Pearson's Chi-squared test; Kruskal-Wallis rank sum test; ^3^ Fisher's exact test. * Number of patients with late follow-up ECG: 917 (13.2%) patients (group 1: 81 (11.4%), group 2: 512 (14.1%), group 3: 334 (12.7%), p=0.004). Minnesota code categories: M1: Q and QS Patterns, M2: QRS Axis Deviation, M3: High Amplitude R Waves, M4: ST Junction (J) and Segment Depression, M5: T-Wave Items, M6: A-V Conduction Defect, M7: Ventricular Conduction Defect, M8: Arrhythmias, M9: ST Segment Elevation: and Miscellaneous Items.

**Supplementary Figures:**

**Supplementary Figure 1:** Daily entry of eligible patients into the study, in the inclusion period from February 17, 2021 to December 15, 2021.
