# Supplementary figures and images for: Clinical and electrocardiographic outcomes evaluated by telemedicine of outpatients with clinical suspicion of COVID-19 treated with chloroquine compounds in Brazil†
Source: Front Cardiovasc Med. 2023 Feb 15;10:1028398. doi: 10.3389/fcvm.2023.1028398 (PMC9978955; doi:10.3389/fcvm.2023.1028398)

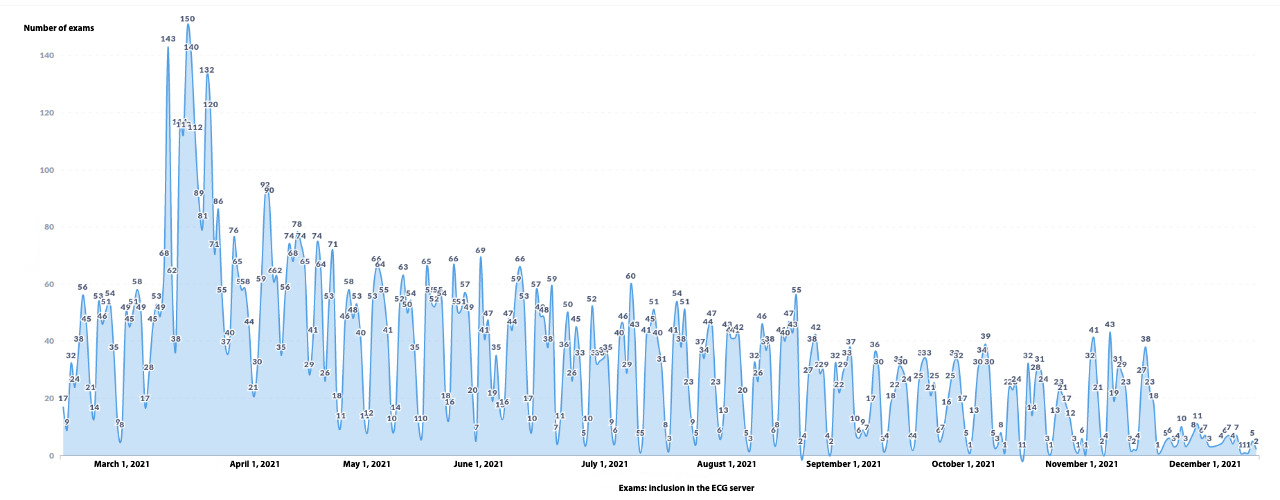

Supplement: Supplementary file 3 [file Image_1.TIF]
